# *Transmembrane serine protease 2* Polymorphisms and Susceptibility to Severe Acute Respiratory Syndrome Coronavirus Type 2 Infection: A German Case-Control Study

**DOI:** 10.3389/fgene.2021.667231

**Published:** 2021-04-21

**Authors:** Kristina Schönfelder, Katharina Breuckmann, Carina Elsner, Ulf Dittmer, David Fistera, Frank Herbstreit, Joachim Risse, Karsten Schmidt, Sivagurunathan Sutharsan, Christian Taube, Karl-Heinz Jöckel, Winfried Siffert, Andreas Kribben, Birte Möhlendick

**Affiliations:** ^1^Department of Nephrology, University Hospital Essen, University of Duisburg-Essen, Essen, Germany; ^2^Institute of Diagnostic and Interventional Radiology and Neuroradiology, University Hospital Essen, University of Duisburg-Essen, Essen, Germany; ^3^Institute for Virology, University Hospital Essen, University of Duisburg-Essen, Essen, Germany; ^4^Center of Emergency Medicine, University Hospital Essen, University of Duisburg-Essen, Essen, Germany; ^5^Department of Anesthesiology and Intensive Care Medicine, University Hospital Essen, University of Duisburg-Essen, Essen, Germany; ^6^Department of Pulmonary Medicine, Ruhrlandklinik, University Hospital Essen, University of Duisburg-Essen, Essen, Germany; ^7^Institute of Medical Informatics, Biometry and Epidemiology, University of Duisburg-Essen, Essen, Germany; ^8^Institute of Pharmacogenetics, University Hospital Essen, University of Duisburg-Essen, Essen, Germany

**Keywords:** transmembrane serine protease 2, coronavirus disease 2019, severe acute respiratory syndrome coronavirus type 2, polymorphism, rs383510, rs2070788, rs12329760, genetic association

## Abstract

The transmembrane serine protease 2 (TMPRSS2) is the major host protease that enables entry of the severe acute respiratory syndrome coronavirus type 2 (SARS-CoV-2) into host cells by spike (S) protein priming. Single nucleotide polymorphisms (SNPs) in the gene *TMPRSS2* have been associated with susceptibility to and severity of H1N1 or H1N9 influenza A virus infections. Functional variants may influence SARS-CoV-2 infection risk and severity of Coronavirus disease 2019 (COVID-19) as well. Therefore, we analyzed the role of SNPs in the gene *TMPRSS2* in a German case-control study. We performed genotyping of the SNPs rs2070788, rs383510, and rs12329760 in the gene *TMPRSS2* in 239 SARS-CoV-2-positive and 253 SARS-CoV-2-negative patients. We analyzed the association of the SNPs with susceptibility to SARS-CoV-2 infection and severity of COVID-19. SARS-CoV-2-positive and SARS-CoV-2-negative patients did not differ regarding their demographics. The CC genotype of *TMPRSS2* rs383510 was associated with a 1.73-fold increased SARS-CoV-2 infection risk, but was not correlated to severity of COVID-19. Neither *TMPRSS2* rs2070788 nor rs12329760 polymorphisms were related to SARS-CoV-2 infection risk or severity of COVID-19. In a multivariable analysis (MVA), the rs383510 CC genotype remained an independent predictor for a 2-fold increased SARS-CoV-2 infection risk. In summary, our report appears to be the first showing that the intron variant rs383510 in the gene *TMPRSS2* is associated with an increased risk to SARS-CoV-2 infection in a German cohort.

## Introduction

The transmembrane serine protease 2 (TMPRSS2) is the major host protease that enables cell entry of several coronaviruses, e.g., HCoV-229E, SARS-CoV, and MERS, respectively (Shulla et al., 2011; Bertram et al., 2013; Shirato et al., 2013). TMPRSS2 is strongly expressed in lung tissue and bronchial transient secretory cells (Lukassen et al., 2020). In a recent study, Hoffmann et al. (2020) demonstrated that SARS-CoV-2 uses the angiotensin-converting enzyme 2 (ACE2) as a receptor for host cell binding, and the viral spike (S) protein is cleaved into S1 and S2 by TMPRSS2 to allow fusion of the viral and cellular membranes.

Single nucleotide polymorphisms (SNPs) in the gene *TMPRSS2* (chromosome 21q22.3, reverse strand) have been associated with susceptibility to and severity of H1N1 or H1N9 influenza A virus infections (Cheng et al., 2015). Currently, SNPs altering gene expression or protein function are frequently discussed in the light of Coronavirus disease 2019 (COVID-19) in literature reviews and in *in silico* analyses utilizing genotyping data from existing databases (Asselta et al., 2020; Barash et al., 2020; Fujikura and Uesaka, 2020; Ghafouri-Fard et al., 2020; Hou et al., 2020; Paniri et al., 2020; Russo et al., 2020; Senapati et al., 2020; Singh et al., 2020; Smatti et al., 2020; Vargas-Alarcón et al., 2020; Zarubin et al., 2020). Three variants, rs2070788, rs383510, and rs12329760, seem to be of particular interest based on preliminary work in the context of other virus infections.

In a study with Chinese patients, a genetic predisposition to severe H1N1 influenza A virus infection was observed for *TMPRSS2* rs2070788 GG genotype (OR: 2.11, 95% CI 1.18–3.77, *p* = 0.01) or G-allele (OR: 1.54, 95% CI 1.14–2.06, *p* = 0.004) carriers (Cheng et al., 2015). The SNP rs2070788 is a G to A transition located in intron 11–12 (c.1171+587C>T) of *TMPRSS2*. Minor allele frequencies (MAFs) for the G-allele are higher in Europeans (0.46) than East Asians (0.36) or Africans (0.27; Clarke et al., 2017). In a lung expression quantitative trait loci (eQTL) analysis, rs2070788 was correlated to differential expression of *TMPRSS2*, with GG genotype carriers showing the highest expression levels (GTEx portal; Irham et al., 2020). The functional basis is still not understood, since rs2070788 is not located in any known regulatory region (ensembl.org, release 102, November 2020).

A second intronic SNP, rs383510 (c.445+1954A>G, intron 5–6), which is in high linkage disequilibrium (LD) with rs2070788 in East Asians (*r*^2^/D' > 0.8) but not Europeans (*r*^2^ = 0.4, D' = 0.7), is located in a putative enhancer region (Cheng et al., 2015). The MAF of the T-allele is about one and a half times higher for Europeans (0.49) than for East Asians (0.36) or Africans (0.33; Clarke et al., 2017). This variant was shown to be an eQTL in the lung with TT genotype carriers showing the highest expression levels (GTEx portal; Irham et al., 2020). Cheng et al. (2015) observed a significant association with H7N9 influenca A virus infection risk for rs2070788 GG or GA genotype carriers (OR: 1.99, 95% CI 1.14–3.48, *p* = 0.02) and rs383510 TT or TC genotype carriers (OR: 2.01, 95% CI 1.15–3.54), *p* = 0.01) in their study comparing the genotype frequencies of Chinese H7N9 influenca A virus infected patients (*N* = 102) to healthy controls (*N* = 106; Cheng et al., 2015).

Because of its functional impact, the missense variant rs12329760 (c.478G>A, p.V160M), which alters the amino acid valine at position 160 to methionine, might as well be of interest with regard to COVID-19. The SNP is located in the scavenger receptor cysteine-rich (SRCR) domain of TMPRSS2, which interacts with external pathogens (Paoloni-Giacobino et al., 1997). The amino acid exchange may create a *de novo* pocket protein (Paniri et al., 2020). It is an eQTL in whole blood with TT genotype carriers showing the highest expression levels (GTEx portal). The MAF of the T-allele in East Asians (0.36) is comparably higher than in Europeans (0.24), South Asians (0.23), or Africans (0.29; Clarke et al., 2017). In prostate cancer, the rs12329760 T-allele is associated with multiple copies of the *TMPRSS2-ERG* gene fusion responsible for shorter survival and higher tumor recurrence (FitzGerald et al., 2008). In a preliminary small scale, whole-exome sequencing study comprising 131 Italian hospitalized COVID-19 patients, Latini et al. (2020) observed a lower rs12329760 T-allele frequency (*p* = 0.02) in the patients compared to corresponding European GnomAD database controls.

Based on the above described associations, we investigated the influence of the variants rs2070788, rs383510, and rs12329760 in the gene *TMPRSS2* on SARS-CoV-2 infection risk and severity of COVID-19 in a German cohort.

## Materials and Methods

### Study Participants, Recruitment, and Outcome of the Patients

The study was conducted following the approval of the Ethics Committee of the Medical Faculty of the University of Duisburg-Essen (20-9230-BO) and in cooperation with the West German Biobank (WBE; 20-WBE-088). Written informed consent was obtained from the study patients.

Enrolment started on March 11, 2020, and ended on September 30, 2020. Patients were initially recruited upon presentation with COVID-19 typical symptoms, i.e., fever, cough, and dyspnea or who were admitted to the hospital with already confirmed SARS-CoV-2 infection. We included 239 SARS-CoV-2-positive patients. Patients were classified as SARS-CoV-2-positive with at least one positive real-time reverse transcription PCR (RT-PCR) test result. Follow-up was completed on October 31, 2020, at which time all patients either were discharged from the hospital as “cured” or had a fatal outcome of the disease. We also studied 253 SARS-CoV-2-negative patients, who presented with COVID-19 typical symptoms, but were tested exclusively negative for SARS-CoV-2 by RT-PCR. These patients were hospitalized at the University Hospital Essen or treated as outpatients, due to other medical conditions. Clinical outcome was defined as follows according to the criteria of the European Center of Disease Prevention and Control (ECDC; 2020) - “serious”: hospitalized patients admitted to an intensive care unit and all cases of COVID-19‑related deaths during the hospital stay; “moderate”: outpatients and all other hospitalized patients. In contrast to the ECDC classification, where patients are counted up to three times, every patient only counted once according to the worst clinical outcome observed during the hospital stay in our study.

### Genotyping of TMPRSS2

PCR was performed with 2 μl genomic DNA and 30 μl *Taq* DNA-Polymerase 2x Master Mix Red (Ampliqon, Odense, Denmark) with the following conditions: initial denaturation 95°C for 5 min; 38 cycles with denaturation 95°C for 30 s; annealing at 60°C (rs12329760) or 65°C (rs2070788 and rs383510, respectively) for 30 s and elongation 72°C for 30 s each; and final elongation 72°C for 10 min. The following oligonucleotide primers were used for the respective PCR reactions: rs2070788 5' [BIO]AGTTTCTGCTGATGAGGAGCC 3', 3'GAAGTGCTTAGTGGCAGGCA 5'; rs383510 5' [BIO]ATGGCTGTGCTTGGGAAATAAC 3', 3' CTTATTTCCTGGCCGGACGC 5'; and rs12329760 5' CGCCCGTAGTTCTCGTTCC 3', 3' TTCGCCTCTACGGACCAAAC 5'. PCR products for rs12329760 were digested with Hpy8I (Thermo Scientific, Dreireich, Germany), and restriction fragments were analyzed by agarose gel electrophoresis. For the various genotypes results from restriction fragment length polymorphism (RFLP)-PCR were validated by Sanger sequencing (Supplementary Figure S1). Genotyping of rs2070788 and rs383510 was performed by Pyrosequencing according to the manufacturers’ instructions (Qiagen, Hilden, Germany). In brief, biotinylated PCR amplicons were immobilized on streptavidin-coated sepharose beads (GE Healthcare, Solingen, Germany) with the vacuum tool of the workstation (Qiagen, Hilden, Germany). In the next step, PyroMark denaturation solution (Qiagen, Hilden, Germany) was used to separate the complementary strand from the biotinylated strand. The biotinylated, single-stranded DNA remains immobilized on the vacuum tool. Finally, the single-stranded DNA was released into the sequencing plate containing 0.3 μM sequencing primer (5' TTTATATCCTTCTCAAAAG 3' for rs2070788 and 5' ACGTGGTGGTGCGGGCCT 3' for rs383510, respectively). After incubation at 80°C for 2 min, the hybridized primer and single-stranded template were incubated with the enzymes DNA polymerase, ATP sulfurylase, luciferase, and apyrase, as well as the substrates adenosine 5' phosphosulfate (APS) and luciferin. Addition of dideoxyribonucleotide triphosphates (ddNTPs) was performed sequentially. Each incorporation event is accompanied by the release of pyrophosphate (PPi) in a quantity equimolar to the amount of the incorporated nucleotide. ATP sulfurylase converts PPi to ATP, which drives luciferase-mediated conversion of luciferin to oxyluciferin that generates visible light. The height of each light signal is proportional to the number of nucleotides incorporated and can be visualized in the Pyrogram. Apyrase continuously degrades unincorporated nucleotides and ATP and when degradation is complete another nucleotide is added. Analyses were performed on the PyroMark Q96 MD instrument (Qiagen, Hilden, Germany).

Transmembrane serine protease 2 is located on the reverse strand, and all polymorphisms are reported on the forward strand in this study.

### Statistical Analyses

Hardy-Weinberg equilibrium (HWE) was calculated using Pearson’s *Χ*^2^ goodness of fit test, and samples were considered as deviant from HWE at a significance level of *p* < 0.05. LD analysis was performed using HaploView V4.2.

For genetic association, we calculated odds ratio (OR) and 95% CI by Fisher’s exact test using Baptista-Pike method for OR, respectively. Values of *p* are reported two-sided and values of *p* < 0.05 were considered significant. Adjustment for multiple comparisons in the genotype-phenotype association analysis was performed using Holm-Bonferroni method (*α* = 0.0167). MVA was performed for both SARS-COV-2 infection risk and COVID-19 severity to estimate independency of the variables age, sex, and *TMPRSS2*, and rs2070788, rs383510, and rs12329760 genotypes by logistic regression (likelihood ratio test, backwards).

The eQTL data described in this manuscript were obtained from the Genotype-Tissue Expression (GTEx) Portal (gtexportal.org; GTEx analysis release V8; dbGaP Accession phs000424.v8.p2) on 01/31/2021.

## Results

From March 11, 2020 to October 31, 2020, we enrolled and studied 239 SARS-CoV-2-positive and 253 SARS-CoV-2-negative patients to determine associations of the SNPs rs2070788, rs383510, and rs12329760 in the gene *TMPRSS2* with susceptibility to SARS-CoV-2 infection and severity of COVID-19. The characteristics and genotypes of SARS-CoV-2-positive and -negative patients are summarized in Table 1. Distribution of sex (*p* = 0.86) and age (*p* = 0.10) was similar in both groups.

**Table 1:** Demographics, transmembrane serine protease 2 (*TMPRSS2*) genotypes, and outcome of the severe acute respiratory syndrome coronavirus type 2 (SARS-CoV-2)-positive and SARS-CoV-2-negative patients.

	SARS-CoV-2-negative (*N* = 253)	SARS-CoV-2-positive (*N* = 239)	Moderate COVID-19 (*N* = 164)	Serious COVID-19 (*N* = 75)
Median age (range) – yrs.	63.0 (22–97)	59.0 (18–99)	57.0 (18–94)	64.0 (26–99)
Male sex – no. (%)	147 (58.1)	141 (59.0)	86 (52.4)	55 (73.3)
*TMPRSS2* rs2070788 G/A				
GG	50 (19.8)	55 (23.0)	40 (24.4)	15 (20.0)
GA	128 (50.6)	118 (49.4)	75 (45.7)	43 (57.3)
AA	75 (29.6)	66 (27.6)	49 (29.9)	17 (22.7)
Minor allele frequency (G)	0.45	0.48	0.47	0.49
GG vs. GA + AA	OR: 1.10, 95% CI [0.75–1.63], *p* = 0.69		OR: 1.45, 95% CI [0.77–2.74], *p* = 0.28	
G vs. A	OR: 1.11, 95% CI [0.78–1.59], *p* = 0.59		OR: 1.06, 95 %CI [0.61–1.82], *p* = 0.89	
*TMPRSS2* rs12329760 C/T				
CC	164 (64.8)	139 (58.2)	91 (55.5)	48 (64.0)
CT	78 (30.8)	84 (35.2)	61 (37.2)	23 (30.7)
TT	11 (4.4)	16 (6.7)	12 (7.3)	4 (5.3)
Minor allele frequency (T)	0.20	0.24	0.26	0.21
TT + CT vs. CC	OR: 1.33, 95% CI [0.92–1.91], *p* = 0.14		OR: 0.70, 95% CI [0.40–1.23], *p* = 0.26	
T vs. C	OR: 1.30, 95% CI [0.85–2.00], *p* = 0.23		OR: 0.76, 95% CI [0.39–1.45], *p* = 0.43	
*TMPRSS2* rs383510 T/C				
TT	57 (22.5)	48 (20.1)	31 (18.9)	17 (22.7)
TC	143 (56.5)	116 (48.5)	81 (49.4)	35 (46.7)
CC	53 (21.0)	75 (31.4)	52 (31.7)	23 (30.7)
Minor allele frequency (T)	0.51	0.44	0.44	0.46
CC vs. TC + TT	OR: 1.73, 95% CI [1.15–2.59], *p* = 0.01^*^		OR: 0.95, 95% CI [0.53–1.72], *p* = 0.88	
C vs. T	OR: 1.29, 95% CI [0.91–1.85], *p* = 0.18		OR: 0.91, 95% CI [0.53–1.57], *p* = 0.78	

Clinical outcome was defined as follows according to the criteria of the European Center of Disease Prevention and Control (ECDC; 2020) - “moderate”: outpatients and hospitalized patients; “serious”: hospitalized patients admitted to an intensive care unit and/or became dependent on mechanical ventilation and all cases of Coronavirus disease 2019 (COVID-19)-related deaths during the hospital stay. Adjustment for multiple comparisons in genotype-phenotype association analysis was performed using the Holm-Bonferroni method (*α* = 0.0167). Significant associations are marked by asterisks (^*^). YRS, years; OR, odds ratio; CI, confidence interval; *p*, *p*-value as calculated from the estimation of the associations of TMPRSS2 genotypes and SARS-CoV-2 infection risk or COVID-19 severity, respectively.

Transmembrane serine protease 2 rs2070788 genotypes were compatible with HWE (*p* = 0.87 and *p* = 0.73, respectively). The MAF of the G-allele in SARS-CoV-2-positive patients (0.48) was similar to the MAF in SARS-CoV-2-negative patients (0.45). For *TMPRSS2* rs12329760, we also did not observe a deviation from HWE for both SARS-CoV-2-negative and -positive patients (*p* = 0.66 and *p* = 0.50, respectively). The MAF of the T-allele was higher in SARS-CoV-2-positive patients (0.24 vs. 0.20). Neither the rs2070788 nor rs12329760 variant was correlated with an increased SARS-CoV-2 infection risk or severity of COVID-19 (Table 1).

The observed genotype frequencies for *TMPRSS2* rs383510 also were consistent with HWE in SARS-CoV-2-positive patients (*p* = 0.80). There was a slight deviation from the HWE in SARS-CoV-2-negative patients (*p* = 0.04). We found 20.1% TT, 48.5% TC, and 31.4% CC genotype carriers in the infected patients. The following genotype frequencies were observed in the non-infected group: 22.5% TT, 56.5% TC, and 21.0% CC. The MAF of the T-allele was higher in the SARS-CoV-2-negative patients (0.51) compared to the MAF in the SARS-CoV-2-positive patients (0.44). Carriers of the CC genotype had a 1.73-fold increased risk to SARS-CoV-2 infection (OR: 1.73, 95% CI 1.15–2.59, *p* = 0.01). We did not observe an association of any allele to severity of COVID-19.

In this study, we did not observe linkage disequilibrium between the alleles of the three loci. The polymorphisms rs2070788 and rs383510 showed highest LD values (*r*^2^ = 0.49, D' = 0.72).

In a multivariable analysis, *TMPRSS2* rs383510 remained an independent predictor for SARS-CoV-2 infection risk (OR: 2.00, 95% CI 1.30–3.08, *p* = 0.002). In a second multivariable analysis, male sex was the only independent predictor for severity of COVID-19 [OR: 2.64, 95% CI (1.44–4.84), *p* = 0.002].

## Discussion and Study Limitations

Currently, the influence of host genetic factors on SARS-CoV-2 infection risk or COVID-19 severity is being investigated in a variety of *in silico* analyses and with targeted or genome-wide sequencing approaches.

Recently, both blood group A and inborn errors of interferon I immunity were identified as risk factors for severity of COVID-19 in genome-wide association studies (Ellinghaus et al., 2020; Zhang et al., 2020).

So far, mainly *in silico* studies have been performed to analyze the influence of variants in the gene *TMPRSS2* with regard to COVID-19 (Asselta et al., 2020; Barash et al., 2020; Fujikura and Uesaka, 2020; Ghafouri-Fard et al., 2020; Hou et al., 2020; Paniri et al., 2020; Russo et al., 2020; Senapati et al., 2020; Singh et al., 2020; Smatti et al., 2020; Vargas-Alarcón et al., 2020; Zarubin et al., 2020).

In a first small-scale study, Latini et al. (2020) observed a significantly lower frequency of rs12329760 T-allele carriers in Italian SARS-CoV-2-positive patients (*N* = 131) compared to a European reference group from the GnomAD database. Interestingly, we found a non-significant higher prevalence of T-allele carriers in the SARS-COV-2-positive patients (0.24) compared to the SARS-CoV-2-negative patients (0.20). This would make sense, if one assumes that T-allele carriers have a higher gene expression (GTEx portal), which could enhance TMPRSS2 activity and so may result in a higher infection susceptibility. Our observations would need to be analyzed in additional patients for this purpose.

This is the first case-control study analyzing the role of *TMPRSS2* rs2070788 with regard to COVID-19. Although it seems plausible from a biological point of view, we did neither observe differences in allele or genotype frequencies between infected and non-infected patients nor different courses of the disease. The influence of this polymorphism has so far only been shown in a study with Chinese H1N1/H7N9 influenza A virus infected patients (Cheng et al., 2015). It cannot be excluded that this variant is relevant in a larger cohort or in other ethnic groups. Since the allele frequencies differ markedly between East Asians (0.36) and Europeans (0.46; Clarke et al., 2017), the latter hypothesis could well play a role. The variant rs2070788 was in high LD with rs383510 in the study with Chinese individuals by Cheng et al. (2015), which we did not observe in our German cohort. These findings fit the LD database records (Clarke et al., 2017) and again underscore the hypothesis that the variant rs2070788 might have a greater significance in other ethnicities.

Remarkably, we found a significant association to a 1.73-fold increased infection risk for *TMPRSS2* rs383510 CC genotype carriers in our German cohort. Since genotyping errors can be excluded we have no explanation for the observation that *TMPRSS2* rs383510 genotypes slightly violated HWE in the SARS-CoV-2-negative but not in the SARS-CoV-2-positive group. This could hypothetically be attributed to an unrecognized selection bias in the first-mentioned group. Multivariable logistic regression analysis confirmed the CC genotype as an independent risk factor for SARS-CoV-2 susceptibility. The mechanism behind our finding remains ambiguous or even counterintuitive. Considering the data from the GTEx database, it appears that the C-allele results in decreased gene expression in the lung (GTEx portal, 2020). Similarly, for H7N9 influenza A virus infected patients, it was observed that TT or TC genotype carriers had a higher infection risk, which seems quite plausible when viewed in conjunction with the expression data (Cheng et al., 2015). The GTEx data are based on post-mortem gene expression analysis in 515 individuals. Further functional studies need to be performed to validate the influence of the rs383510 polymorphism on mRNA or protein expression and function especially in the context of COVID-19. As mentioned above, our findings cannot be transferred to populations with different ethnicities and need to be validated in larger cohorts as well.

Regarding a genetic predisposition to a serious course of COVID-19, we did not observe any association with the analyzed *TMPRSS2* variants. On the one hand, this could be due to the biological function of *TMPRSS2* as a mediator of cell entry for SARS-CoV-2, so that differences in protein function might only influence the infection risk, rather than the course of the disease. On the other hand, the sample size of our cohort with “serious” COVID-19 was relatively small (*N* = 75). Further analyses are needed to conclusively clarify the influence of *TMPRSS2* variants on disease severity.

In conclusion, for the first time, we report here an association of *TMPRSS2* rs383510 CC genotype with SARS-CoV-2 infection susceptibility. Neither *TMPRSS2* rs2070788 nor rs12329760 polymorphisms were related to SARS-CoV-2 infection risk or severity of COVID-19 in our German cohort. Before universally being acceptable, the role of variants in the gene *TMPRSS2* in SARS-CoV-2 infections still needs to be elucidated in larger cohorts encompassing various ethnicities.

## Data Availability Statement

The datasets presented in this study can be found in online repositories. The names of the repository/repositories and accession number(s) can be found in the article/Supplementary Material.

## Ethics Statement

The studies involving human participants were reviewed and approved by the Ethics Committee of the Medical Faculty of the University of Duisburg-Essen (20-9230-BO). The patients/participants provided their written informed consent to participate in this study.

## Author Contributions

KrS: resources, methodology, data curation, formal analysis, validation, and writing – review and editing. KB: data curation and investigation. CE: resources and investigation. UD, DF, FH, JR, KaS, SS, and CT: investigation. K-HJ: investigation and validation. WS and AK: conceptualization, validation, supervision, and writing – review and editing. BM: conceptualization, resources, methodology, formal analysis, investigation, supervision, data curation, funding acquisition, project administration, visualization, writing – original draft, and writing – review and editing. All authors contributed to the article and approved the submitted version.

### Conflict of Interest

The authors declare that the research was conducted in the absence of any commercial or financial relationships that could be construed as a potential conflict of interest.
