# Supplementary material for: Transmembrane serine protease 2 Polymorphisms and Susceptibility to Severe Acute Respiratory Syndrome Coronavirus Type 2 Infection: A German Case-Control Study
Source: Front Genet. 2021 Apr 21;12:667231. doi: 10.3389/fgene.2021.667231 (PMC8097083; doi:10.3389/fgene.2021.667231)
Supplement: Supplementary file 1 [file Data_Sheet_1.PDF]

Supplementary data

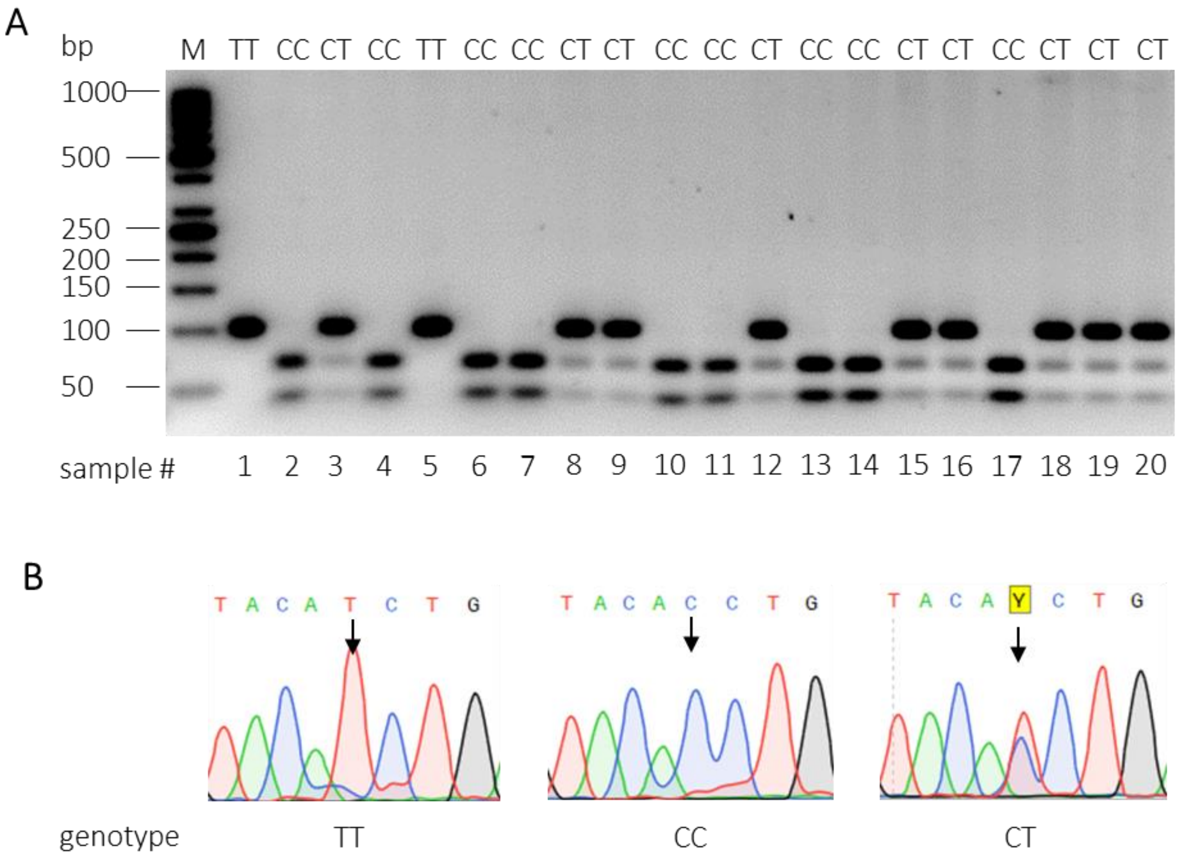

Figure S1. Comparison of results from RFLP-PCR and Sanger sequencing for *TMPRSS2* rs12329760 genotyping. A) Agarose gel analysis of *TMPRSS2* rs12329760 RFLP-PCR products. PCR products were digested with Hpy8I (GTN^NAC). Restriction site is present in C-allele carriers. Fragment sizes are 100 bp for TT genotype, 100 + 65 + 35 bp for CT genotype and 65 + 35 bp for CT genotype, respectively. **B)** Matching electropherograms for samples #1-3. Arrow indicates the rs12329760 SNP position.

Abbreviations: M = Marker (GeneRuler 50 bp DNA ladder, Thermo Scientific, Waltham, Massachusetts, USA), bp = base pairs, Y = pyrimidine (C/T).
